# Studying Perfusion Effects on Heat Transfer in Tissue-Mimicking Phantoms for Cardiac Ablation: A Preliminary Experimental Investigation

**DOI:** 10.11159/jffhmt.2026.007

**Published:** 2026-01-26

**Authors:** Nooruldeen Essam Mustafa, Brett Wrubleski, Cristian A. Linte, Satish G. Kandlikar

**Affiliations:** 1 Rochester Institute of Technology, Department of Mechanical Engineering, 1 Lomb Memorial Dr. Rochester, NY 14623 USA; 2 Rochester Institute of Technology, Department of Mechanical Engineering, 1 Lomb Memorial Dr. Rochester, NY 14623 USA

**Keywords:** Bioheat transfer, tissue-mimicking phantom, radiofrequency ablation, cardiac arrhythmia, perfusion cooling

## Abstract

Cardiac arrhythmias are commonly treated using radiofrequency ablation, a minimally invasive technique that has become standard practice. Despite its widespread use, the mechanisms of heat transfer through cardiac tissue during ablation and the factors determining lesion geometry remain incompletely understood. This study investigates the thermal behaviour of tissue-mimicking phantoms with and without perfusion channels to quantify the perfusion-mediated cooling effects present in cardiac ablation. A controlled experimental apparatus was engineered to simulate the *in vivo* thermal conditions of cardiac tissue. The experiments were performed in a water bath at 37°C, simulating body temperature, and a copper heating element manufactured with high accuracy was used instead of a radiofrequency ablation electrode. Several thermocouples were inserted into the phantom to record temperature changes over time and space, allowing for accurate monitoring of temperature fluctuations. The experiments were carried out in polyacrylamide phantoms containing microchannels, and the flow rate through the channels was varied between 15 and 25 mL/min. The temperature of the heating element was varied between 324 K and 364 K, a range that is relevant for radiofrequency ablation applications. The temperature distribution was affected by the flow in the microchannels, as perfusion reduced the temperature increase in the phantom. The cooling effect exhibited spatial heterogeneity, with temperature distribution varying significantly based on position relative to the perfusion channels. The downstream measurement points (downstream according to the direction of flow in the microchannels) exhibited a higher mean temperature decrease (22%) than the upstream points (10%), and perfusion decreased the temperature gradient between these two regions, homogenizing the heat distribution. These findings provide quantitative benchmarks for incorporating perfusion effects into computational ablation models, potentially enabling more accurate lesion prediction and improved procedural planning in clinical practice.

## 1 INTRODUCTION

### 1.1 Bioheat Transfer in Medical Applications

Heat transfer is fundamental to maintaining physiological balance during everyday bodily functions and medical interventions [1]. Bioheat transfer follows the basic principles of heat transfer physics, but is complicated by blood perfusion, metabolic heat generation, and the biological tissues’ heterogeneous, anisotropic nature [2]. Understanding these complex mechanisms is crucial for optimizing medical treatments, particularly thermal therapies, where precise temperature control is essential for both efficacy and safety [3].

The clinical motivation for this work is particularly relevant for the treatment of atrial fibrillation (AF), the most common cardiac arrhythmia. A primary therapeutic target during AF ablation is the ostia of the pulmonary veins, which are located within the moderately vascularized atrial wall [4], [5]. The success of ablation procedures in these regions is critically challenged by heat sink effects from adjacent blood flow, which can cool the tissue and prevent the formation of transmural, durable lesions [6], [7]. While computational models have attempted to predict these effects [7], [8], current ablation lesion assessment tools remain limited by thermal models that may not fully capture patient-specific perfusion patterns [7], [10]. This gap between simplified models and physiologically realistic models may contribute to inadequate prediction of lesion quality and geometry and consequently to the delivery of incomplete lesions that lead to subpar procedure outcomes and need for repeat ablation interventions [9]. Our experimental framework directly addresses this critical perfusion factor by providing quantitative benchmarks for perfusion cooling that can be integrated into next-generation lesion prediction models, capturing the impact of tissue perfusion and potentially improving the success of ablation procedures on the first attempt. This gap hinders the optimization of ablation parameters—such as power, duration, and contact force—necessary to achieve procedural success in high-flow environments. Therefore, developing a controlled experimental framework to quantify perfusion-mediated cooling is a crucial step toward improving clinical outcomes.

The study of bioheat transfer has evolved significantly since the introduction of the Pennes bioheat equation in 1948 [10], which remains the foundation for most bioheat transfer models [5, 6, 7]. This equation, shown below, accounts for conduction, perfusion, and metabolic heat generation:

(1) $$\rho c\frac{\partial T}{\partial t}=k\nabla^{2}T+\omega \rho_{b}c_{b}\left(T_{b}-T\right)+Q_{m}+Q,$$

where ρ is tissue density (kg/m^3^), c is the specific heat capacity of tissue (J/*kg* · *K*), T is tissue temperature (K), k is thermal conductivity (W/m·K), ω_b_ is the blood perfusion rate (m^3^/s·m^3^), c_b_ is blood specific heat (J/kg · K), T_b_ is arterial blood temperature (K), and Q_m_ is metabolic heat generation (W/m^3^).

For applications involving external energy sources, such as radiofrequency ablation, an additional heat source term Q_e_ is added to account for the electromagnetic energy deposition [14]:


(2) $$\rho c\frac{\partial T}{\partial t}=k\nabla^{2}T+\omega \rho_{b}c_{b}\left(T_{b}-T\right)+Q_{m}+Q_{e}$$

The Pennes equation remains a foundational yet simplified framework, primarily due to its assumptions of uniform tissue properties and a lumped parameter approach to perfusion [15]. To closely mimic real tissue, more advanced models have emerged. These include the structurally detailed Weinbaum-Jiji equation, which accounts for the directional nature of vasculature [16], and fractional calculus methods capable of modelling the non-standard heat transfer observed in complex, heterogeneous biological tissues [17], [18].

### 1.2 Cardiac Ablation

Radiofrequency (RF) ablation is currently the preferred method for treating cardiac arrhythmias, offering a less invasive approach compared to medication or open-heart surgery [19]. The procedure involves delivering alternating current through an electrode catheter positioned at the arrhythmogenic site within the heart. The RF current (typically 500–750 kHz) generates an electromagnetic field that causes rapid oscillation of charged molecules in the tissue, producing frictional heating [20]. When tissue temperature reaches approximately 50°C, irreversible protein denaturation occurs, creating a non-conductive lesion that interrupts abnormal electrical pathways [21].

The efficacy of RF ablation depends on creating lesions of sufficient depth and volume to permanently interrupt arrhythmogenic circuits while avoiding complications such as steam pops, char formation, or damage to adjacent structures [22]. This delicate balance requires precise characterization and control of the heat transfer mechanisms in the tissue [11], [13].

Several factors influence lesion formation during cardiac RF ablation. One critical factor is the catheter-tissue contact force, where a higher contact force enhances energy transfer efficiency, resulting in larger lesions [23]. Additionally, the power and duration of RF energy application play significant roles, as increased power and longer durations typically produce larger lesions but also elevate the risk of complications [24]. Saline irrigation at the electrode-tissue interface is another important consideration, as it prevents excessive surface heating, thereby facilitating deeper lesion formation [25]. Furthermore, intrinsic tissue properties, such as thermal conductivity, electrical impedance, and tissue thickness, significantly influence the distribution of energy within the tissue [26]. Lastly, blood flow contributes to lesion formation through convective cooling, as circulating blood can substantially influence temperature distribution and thus impact lesion characteristics [9].

Of these factors, blood perfusion presents challenges for both modeling and experimental investigation due to its complex and dynamic nature. Blood flow acts as a heat sink, removing thermal energy from the ablation site through convective cooling [27]. This effect is especially pronounced in the highly vascularized myocardium, where coronary perfusion can significantly alter temperature distributions and lesion geometry [25]. As such, there is an unmet need to assess the effect of tissue perfusion on the heat transfer process by experimentally quantifying the tissue temperature in response to the presence and extent of vessel perfusion, These findings can then be incorporated into ablation models that could be used to predict and characterize ablation lesion geometry and quality, toward ensuring complete tissue necrosis post-ablation.

### 1.3 Tissue-Mimicking Phantoms for Thermal Studies

Tissue-mimicking phantoms are essential for investigating bioheat transfer phenomena in controlled laboratory settings. These phantoms are designed to replicate biological tissues’ physical, thermal, and electrical properties, enabling systematic studies that would be difficult or ethically problematic *in vivo* [28].

For cardiac ablation research, ideal phantoms should possess thermal conductivity (0.49–0.57 W/m·K), specific heat capacity (3500–4200 J/kg·K), and electrical conductivity (0.6–0.7 S/m), similar to myocardial tissue [26]. Additionally, they should maintain stable properties under heating and allow for reproducible experimental conditions.

Recent advances in phantom development include:

1. **Polyacrylamide-based formulations** that closely match the thermal properties of cardiac tissue [29].
2. **Thermochromic materials** that change color at specific temperature thresholds, providing a visual indication of ablation zones [30].
3. **Flow-enabled phantoms** with embedded channels to simulate blood perfusion [31].
4. **Multi-component designs** that incorporate different materials to represent the heterogeneity of cardiac tissue [32].

Among these advanced phantom types, polyacrylamide hydrogel was selected for this study because of its superior material stability under thermal loading, its tunable mechanical properties, which allow for precise embedding of microchannels, and its demonstrated ability to maintain consistent thermal characteristics—key requirements for reproducible ablation experiments [28], [33].

While the Pennes bioheat equation provides a foundational model, its limitations become particularly apparent in a cardiac context. It fails to capture the dynamic, pulsatile nature of coronary perfusion, the strong anisotropy of myocardial tissue due to its fibrous structure, and the complex three-dimensional heat sink effects created by the branching coronary vasculature [34]. This gap between simplified models and physiological reality underscores the critical need for experimental data derived from well-characterized phantoms to validate and refine next-generation computational frameworks for predicting ablation outcomes.

### 1.4 Research Objectives

Despite substantial progress in computational modelling [11], [12], [13] and phantom development, the effects of perfusion on heat transfer from cardiac RF ablation remain unclear. Experimental validation of theoretical models is essential for improving the accuracy of lesion prediction algorithms and optimizing ablation parameters [12], [24]. The current study aims to address this by:

1. Quantifying the cooling effect of simulated blood perfusion in tissue-mimicking phantoms during controlled heating.
2. Investigating the relationship between perfusion rate and temperature distribution within the phantom.
3. Examining the spatial variation in cooling effects relative to perfusion channels.

## 2 RESEARCH METHOD

The following section details the theoretical framework, experimental apparatus, and measurement protocols employed to investigate perfusion-mediated cooling effects across varying flow rates and heating intensities.

### 2.1 Theoretical Framework

The experimental investigation in this study is founded on the principles of transient heat transfer in biological tissues. The modified Pennes bioheat equation can describe the temperature distribution within the tissue-mimicking phantom [2]:

(3) $$\rho c\frac{\partial T}{\partial t}=k\nabla^{2}T+\omega \rho_{f}c_{f}\left(T_{f}-T\right)+Q$$

This formulation integrates three principal energy transport mechanisms: conductive heat transfer through the phantom material, convective heat exchange resulting from fluid perfusion, and an external heat input term accounting for applied energy. By incorporating these elements, the modified Pennes equation can accurately predict changes in tissue temperature or those of a phantom model during experiments. Under these conditions, the effective thermal conductivity (*k*_*eff*_), the volume of the perfused phantom can be estimated using the following relationship [15]:

(4) $$k_{\text{eff}}=k+\omega \rho_{f}c_{f}\frac{L^{2}}{\pi^{2}},$$

where L is the characteristic length scale of the perfusion channels. The local temperature increase within the phantom during heating approximates the solution to a one-dimensional heat conduction equation under a constant heat flux boundary condition [35]:

(5) $$T(x)=T_{0}+\frac{q}{k}x$$

Under steady-state conditions, this results in a linear temperature increase with distance from the heat source, assuming a constant applied heat flux. The magnitude of this temperature rise is governed by both the applied thermal energy and the thermal conductivity of the material.

### 2.2 Experimental Setup

**Figure 1:**
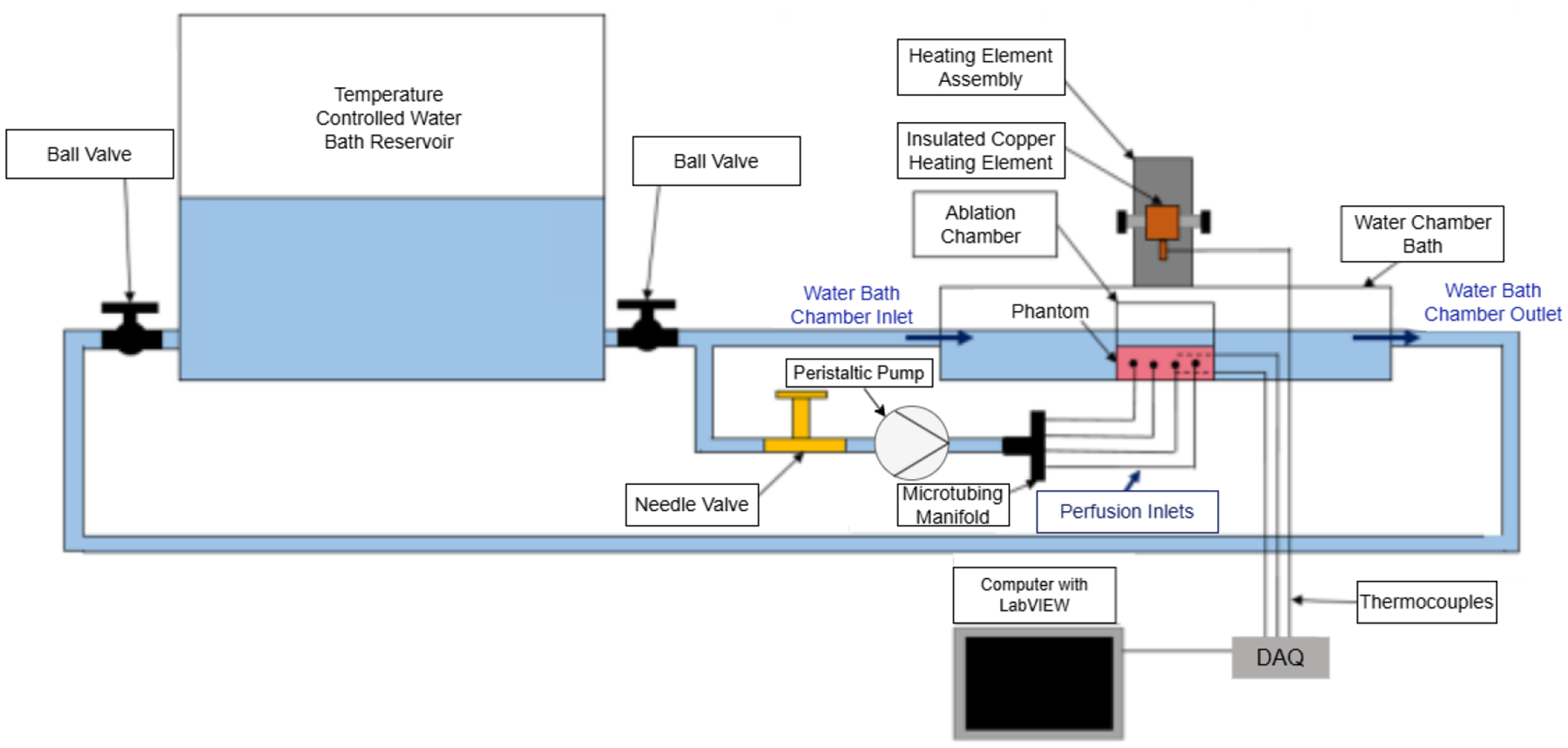
Full Schematic of the setup for perfused and non-perfused phantoms, including ablation chamber, peristaltic pump, pipe flow controls, chiller reservoir, and thermocouples for temperature measurements. Adapted from Cappon [36].

The experimental configuration incorporated six primary subsystems: a recirculating chiller unit, a temperature-controlled water bath, a peristaltic pump for perfusion simulation, a copper heating element, a custom ablation chamber, and a computerized data acquisition system. Fig. 1 shows a schematic of the whole setup [36].

The temperature control system utilizes a VWR Recirculating Chiller to maintain a constant temperature. The chiller maintains a steady temperature by pumping cooled or heated water through the water bath and the connected tubing system. An 8-liter reservoir ensures thermal stability throughout the experiment, preventing significant temperature fluctuations due to heat flux from the ablation process. The water temperature was maintained at 37 ± 0.5°C to simulate physiological conditions within the heart chambers.

The water bath features a custom-designed acrylic chamber with a volume of 1.5 liters. The inlet and outlet pipes from the chiller are strategically positioned at opposite sides of the chamber to create a uniform flow pattern across the bath, ensuring consistent temperature distribution. The chamber is filled to a standardized 70% fill height (approximately 1.05 L) to maintain consistent experimental conditions across trials.

#### 2.2.1 Heating Element and Control System

A precision-machined copper heating element (thermal conductivity: 385 W/m·K) with a diameter of 4 mm was used to simulate the RF electrode. The heating element is mounted on a rack-and-pinion system that allows for precise vertical positioning with an accuracy of ±0.1 mm. This system ensures consistent contact between the heating element and the phantom surface across experimental trials.

The heating element is thermally isolated from the mounting apparatus using Gerolite (thermal conductivity: 0.27 W/m·K) and fiberglass insulation to minimize heat loss through the mounting structure. A K-type thermocouple is embedded within the heating element 1 mm from the contact surface to monitor its temperature throughout the experiment.

Power is supplied to the heating element using a programmable DC power supply (BK Precision 9130B) capable of delivering constant voltage with ±0.1% regulation. The power supply was calculated to provide precise voltage levels of 20V, 25V, and 30V, corresponding to power outputs of approximately 4W, 6.25W, and 9W, respectively.

#### 2.2.2 Perfusion Simulation System

Simulated blood perfusion was achieved using a Cole-Parmer Masterflex peristaltic pump (Model L/S 7550–30), which delivered precise flow control with an accuracy of ±0.5%. Medical-grade silicone tubing with an internal diameter of 1.0 mm connected the pump to the phantom, replicating the scale of small coronary vasculature. The perfusion circuit incorporated a needle valve for flow regulation and a flow meter for verification. Experiments employed three standardized flow rates—15, 20, and 25 mL/min—spanning the physiological range observed in myocardial tissue [37].

#### 2.2.3 Ablation Chamber and Phantom Housing

**Figure 2:**
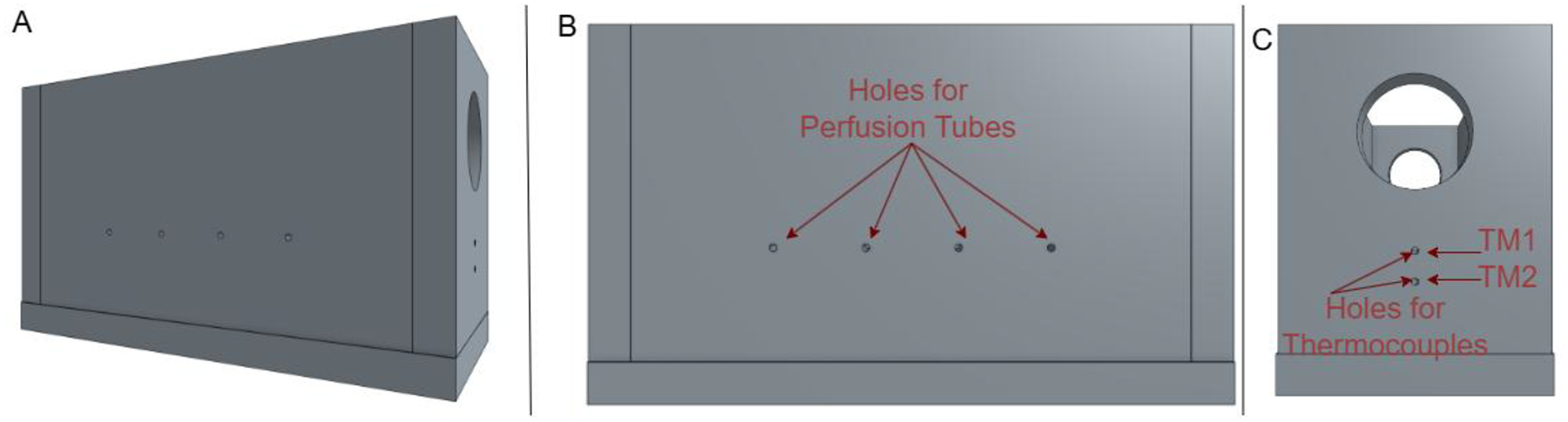
CAD model of the ablation chamber, showing (A) complete visualization, (B) a side profile with holes for perfusion pipes, and (C) a front profile displaying holes for thermocouples.

A custom-designed acrylic ablation chamber houses the tissue-mimicking phantom during experiments. The chamber features precision-drilled holes for inserting perfusion tubing and thermocouples, as shown in Fig. 2.

The chamber dimensions (50 mm × 50 mm × 30 mm) were designed to minimize boundary effects while maintaining a manageable phantom size. The chamber is secured to the water bath using a double-sided adhesive, ensuring consistent positioning across experimental trials.

#### 2.2.4 Tissue-Mimicking Phantom Preparation

Polyacrylamide-based phantoms were fabricated using an optimized formulation designed to replicate the thermal properties of cardiac tissue. The mixture consisted of 80% deionized water, 15% acrylamide/bis-acrylamide solution (30% concentration), 4% ammonium persulfate (10% solution), and 1% tetramethylethylenediamine (TEMED) by weight. This composition produced phantoms exhibiting thermal characteristics closely aligned with myocardial tissue: thermal conductivity of 0.52 ± 0.03 W/m·K, specific heat capacity of 3900 ± 200 J/kg·K, and density of 1050 ± 20 kg/m^3^ [26].

Perfused phantom configurations incorporated silicone microtubing (1.0 mm ID, 1.5 mm OD) positioned horizontally 5 mm beneath the surface. The tubing followed a parallel arrangement with consistent 5 mm inter-channel spacing to simulate distributed vascular structures.

#### 2.2.5 Temperature Measurement System

Thermal monitoring utilized calibrated Omega K-type thermocouples featuring a 0.5 mm diameter. The perfusion channels, consisting of 1.0 mm inner diameter silicone tubing with 1.5 mm outer diameter, were embedded horizontally at a centerline depth of 5 mm below the phantom surface. Two designated sensors (TM1 and TM2) were strategically positioned to capture temperature profiles on either side of these channels. TM1 was positioned 3 mm beneath the surface, placing it approximately 1.25 mm above the upper boundary of the perfusion channels in the upstream heat flux path. TM2 was placed 7 mm deep, positioning it approximately 1.25 mm below the lower boundary of the channels in the downstream heat flux path. This symmetric arrangement ensures that both thermocouples are equidistant from the perfusion channel boundaries while capturing the directional effects of convective cooling. The thermocouples were positioned with a 0.5 mm lateral offset to prevent thermal interference while maintaining proximity to the central heating axis. They were secured using a silicone sealant before phantom polymerization to ensure stable positioning throughout the experimental trials.

Temperature data was acquired using a National Instruments cDAQ-9174 chassis with an NI-9213 thermocouple input module, which provides 24-bit resolution and sampling at 10 Hz. Data acquisition and processing were performed using a custom LabVIEW program that applied calibration corrections and recorded time-stamped temperature values.

### 2.3 Experimental Procedure

The polyacrylamide phantoms were prepared for each experimental condition according to the standardized formulation described in Section 2.4.5, with polymerization completed 24 hours before testing to ensure complete gel formation and material stabilization. The phantom was mounted on the ablation chamber in a water bath using double-sided adhesive to hold it securely during experiments. The water bath was then filled with deionized water to 70% of its capacity, and a chiller was activated to maintain the water at a constant physiological temperature of 37°C. The peristaltic pump was set to the desired flow rate for perfusion trials: 15, 20, or 25 mL/min. Once the system reached steady state, the power supply was adjusted to the specified voltage (20, 25, or 30V), allowing the copper heating element to achieve thermal equilibrium for approximately ten minutes.

The temperature of the heating element was continuously monitored using a thermocouple to ensure precise control. Data acquisition was managed using a custom LabVIEW program initiated with a sampling rate of 10 Hz. Before heating, baseline temperature readings were recorded for 30 seconds to establish initial conditions. The heating phase commenced as the rack and pinion system gently lowered the heating element until it contacted the phantom surface, a process confirmed by a slight increase in resistance on the power supply. The heating element remained in contact with the phantom for exactly 125.6 seconds, a duration determined by collecting 1,256 data points at a 10 Hz sampling rate, which provided sufficient temporal resolution to capture the transient thermal response while achieving quasi-steady-state conditions. Temperature data from all thermocouples were continuously collected throughout this period. Following the heating period, the cooling phase was initiated upon retraction of the heating element from the phantom surface. Following each trial, the system underwent a 30-minute re-equilibration period to return to baseline thermal conditions. The use of fresh phantoms for each experimental condition eliminated concerns about thermal degradation, changes in water content, or alterations in mechanical properties resulting from repeated heating cycles, ensuring that observed differences between trials could be attributed solely to the tested variables of perfusion rate and probe temperature.

### 2.4 Calibration and Uncertainty Analysis

#### 2.4.1 Thermocouple Calibration

The Omega K-type thermocouples used for temperature measurement were calibrated using an Omega Calibration Cell with an NIST-traceable reference thermometer. Calibration was performed in 10 K increments over the temperature range of 293 K to 353 K, encompassing the full range of temperatures expected during the experiments.

Three calibration cycles were performed for each thermocouple, and the data were fitted using least-squares regression to determine the calibration equation. These calibration equations were implemented in the LabVIEW data acquisition software to provide real-time temperature correction.

Despite this calibration, the manufacturer-specified uncertainty for K-type thermocouples is ± 2.2°C or ± 0.75% of the measured value, whichever is greater [38]. Our calibration procedure reduced this uncertainty to approximately ± 0.5°C over the experimental temperature range.

#### 2.4.2 Flow Rate Calibration

The peristaltic pump was calibrated by collecting and weighing the discharged water over a measured period. Calibration was performed at the three flow rates used in the experiments (15, 20, and 25 mL/min). The calibration procedure was repeated five times for each flow rate, yielding an uncertainty of ±0.3 mL/min.

#### 2.4.3 Thermocouple Positional Uncertainty

A significant limitation of this setup is the difficulty in precisely determining thermocouple positions after the phantom material has solidified. Although the thermocouples are initially placed where intended, small movements can occur as the material settles. As a result, their final positions could be somewhat uncertain, as once the material has fully hardened, the thermocouple locations cannot be checked or adjusted. Hence, the exact depth and orientation of each thermocouple remain somewhat uncertain, with positional variability potentially causing temperature measurement errors of up to ±0.4°C.

To assess how the thermocouple positioning uncertainty impacts our key finding of differential upstream/downstream cooling, we analyzed the potential bias. Given ± 0.5 mm thermocouple positional uncertainty relative to the 1.5 mm channel outer diameter, the thermocouple-to-channel distance could vary by up to 40% (from 1.25 mm nominal to 0.75–1.75 mm actual). While we cannot precisely quantify the temperature impact without additional experiments at known positions, our uncertainty analysis suggested an end-ablation temperature difference of 0.5 K. Moreover, the distal thermocouple location (at TM2) experienced approximately twice the cooling effect experienced by the proximal thermocouple (at TM1), across all trials, suggesting that our findings are robust and the thermocouple positioning uncertainty does not lead to significant end-ablation temperature variations. As such, even if thermocouple positioning errors would lead to a perceived reduced cooling advantage downstream of the heat source, the physical principle of directional heat sink effects remains valid. Nevertheless, the limitation associated with thermocouple positioning uncertainty underscores the need for improved techniques to track or confirm thermocouple placement as part of our future research.

#### 2.4.4 Combined Uncertainty Analysis

The combined standard uncertainty in temperature measurements was calculated using the root sum of squares method, considering contributions from thermocouple calibration, data acquisition system, and positional uncertainty:

(6) $$u_{c}(T)=\sqrt{u_{\text{cal}}^{2}+u_{\text{daq}}^{2}+u_{\text{pos}}^{2}}$$

where *u*_*cal*_ is the calibration uncertainty, *u*_*daq*_ is the data acquisition system uncertain, and *u*_*pos*_ is the positional uncertainty.

This analysis yielded a combined standard uncertainty of ±0.7 °C for the temperature measurements. For temperature difference measurements, the uncertainty was reduced to ± 0.5°C due to correlated errors.

#### 2.4.5 Experimental Variability

To assess the reproducibility of the experimental results, each condition was tested three times. The standard deviation of the maximum temperature rise across these three repetitive measurements was calculated, yielding an average experimental variability of ± 0.3°C.

## 3 RESULTS

The following section presents temperature data from experiments conducted at probe temperatures ranging from 324 K to 364 K, with non-perfused trials at 324 K, 344 K, 346 K, and 362 K, and perfused trials using flow rates of 15–25 mL/min at 325 K, 344 K, 346 K, and 364 K. All experiments maintained baseline conditions at 37°C (310 K) in the water bath, with data collected over 125.6 seconds.

### 3.1 Non-Perfused Phantom Results

Initial experiments were conducted on non-perfused phantoms to establish baseline thermal behaviour and validate the experimental setup. Under different heating conditions, these trials investigated the temperature response at two measurement points (TM1 and TM2).

#### 3.1.1 Temperature Evolution Under Different Heating Conditions

**Figure 3:**
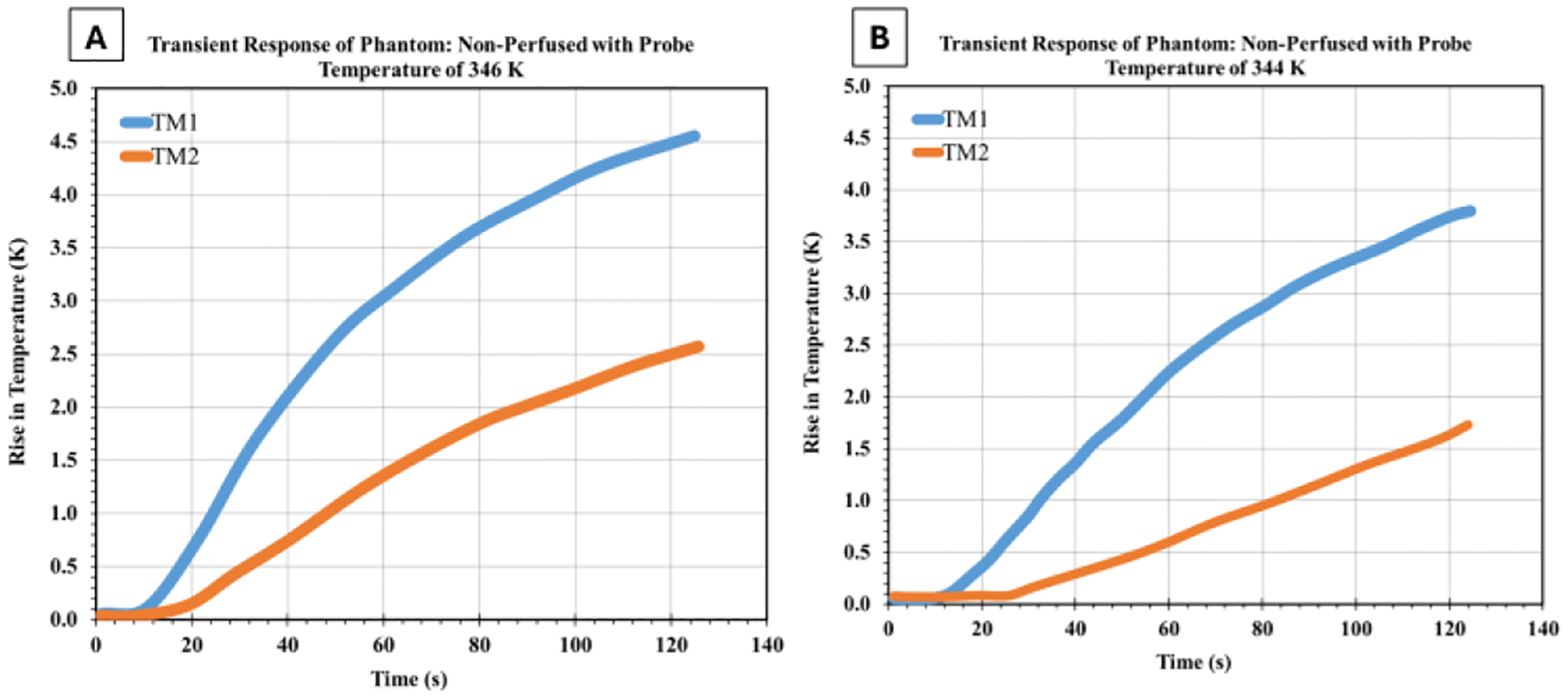
Transient heating behaviour of the non-perfused phantom at probe temperatures of (a) 346 K and (b) 344 K. The rise in temperature at TM1 and TM2 indicates a faster and larger response at the location closer to the probe (TM1), while TM2 shows a more gradual increase.

Figure 3 presents the temperature evolution at TM1 and TM2 during heating with an element temperature of 346 K and 344 K. This trial serves as a baseline for comparison with other experimental conditions.

As shown in Fig. 3a, the temperature at TM1 (3 mm below the surface) increased by 4.5 K over the 125-second heating period, while TM2 (7 mm below the surface) showed a more modest increase of 2.5 K. The final temperature difference between TM1 and TM2 was 2.0 K, reflecting the temperature gradient established within the phantom.

The temperature rise exhibited the characteristic non-linear profile expected from transient heat conduction theory, with a rapid initial increase followed by a gradually decreasing temperature change rate as thermal equilibrium approached.

#### 3.1.2 Effect of Heating Element Temperature

**Figure 4:**
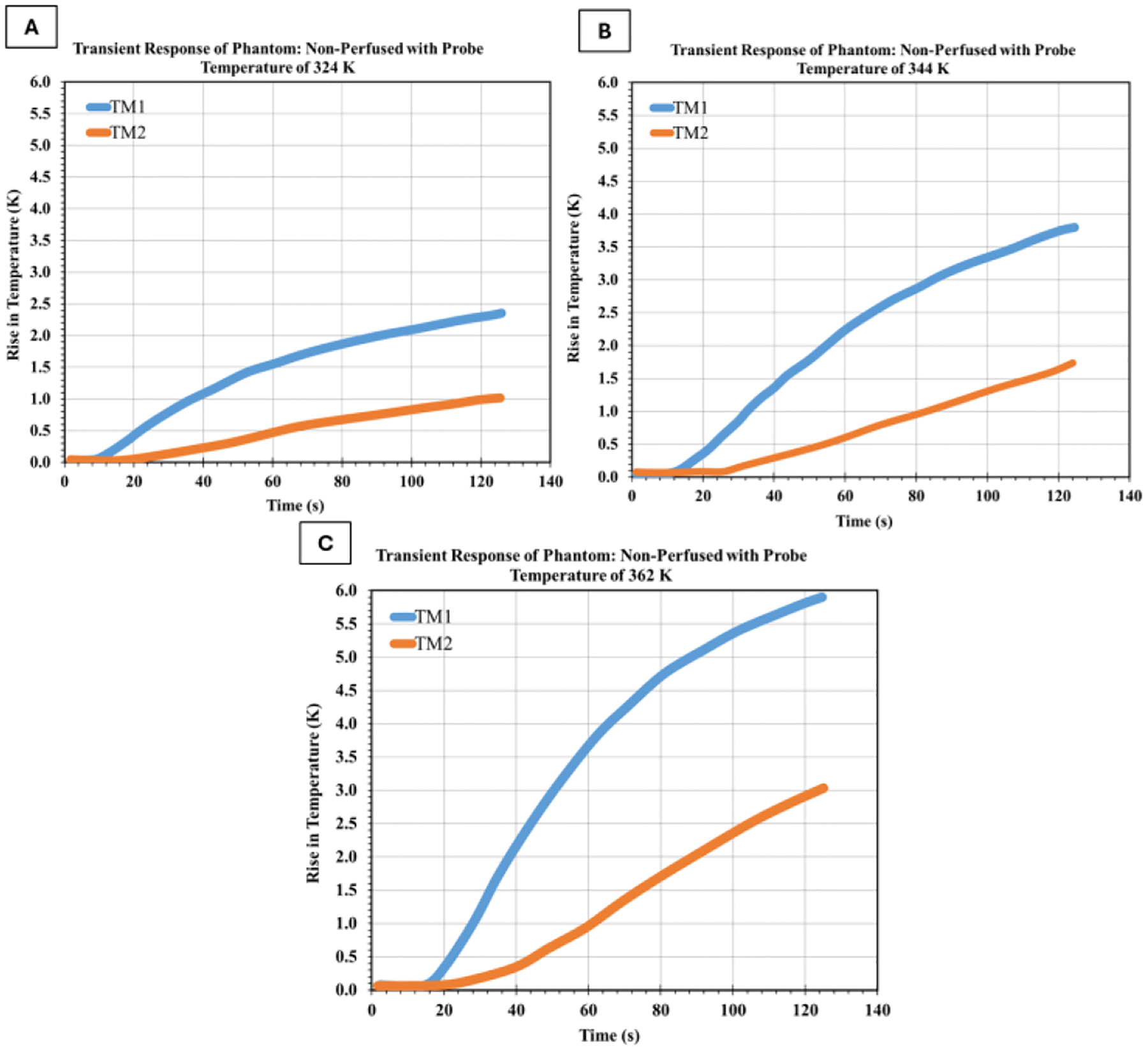
Transient thermal response of the non-perfused tissue-mimicking phantom subjected to different probe temperatures. The temperature rise was monitored at the two thermocouple locations (TM1 and TM2) for probe temperatures of (a) 324 K, (b) 344 K, and (c) 362 K.

Experiments were conducted at three different heating element temperatures: 324 K, 344 K, and 362 K to investigate the relationship between heating element temperature and phantom thermal response. The results are presented in Fig. 4. These results reveal a clear relationship between heating element temperature and temperature rise within the phantom. As the heating element temperature increased from 324 K to 362 K, the maximum temperature rise at TM1 increased from 2.4 K to 5.9 K, while at TM2 it increased from 0.9 K to 3.0 K. The temperature gradient between measurement points increased proportionally with probe temperature, indicating consistent heat transfer characteristics across the tested range.

### 3.2 Perfused Phantom Results

The primary objective of this study was to quantify the effect of perfusion on heat transfer within tissue-mimicking phantoms. Experiments were conducted with perfused phantoms under various flow rates and heating conditions, and the results were compared to the corresponding non-perfused trials using the same phantom in identical positioning.

#### 3.2.1 Perfusion Effects

Experiments were conducted at various heating element temperatures, while maintaining a constant flow rate of 25 mL/min, to investigate the interaction between heating element temperature and perfusion effects.

**Figure 5:**
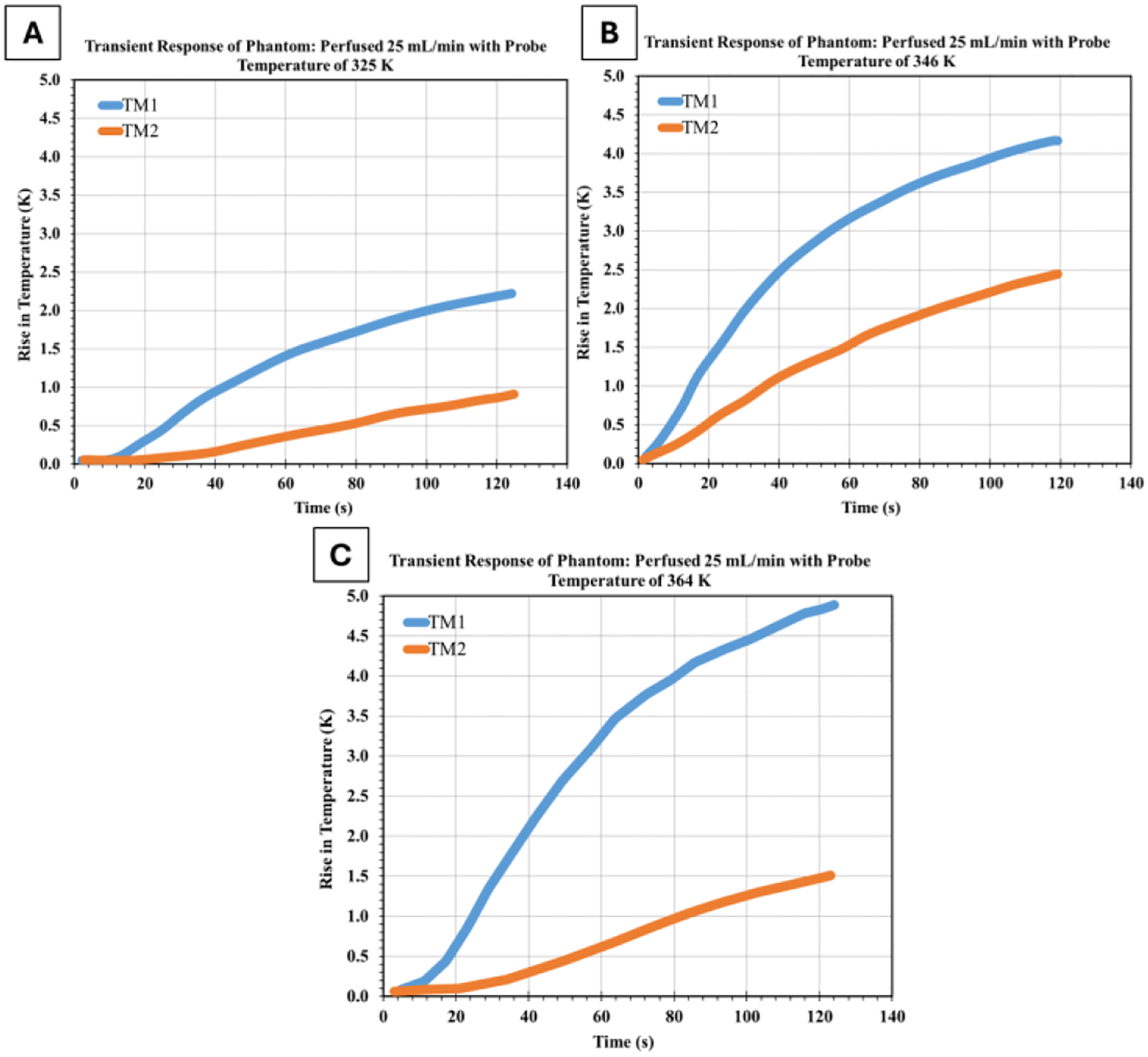
Transient thermal response of perfused tissue-mimicking phantoms at probe temperatures of (a) 325 K, (b) 346 K, and (c) 364 K while maintaining a constant perfusion rate of 25 mL/min.

Figure 5 presents the temperature evolution in a perfused phantom with a flow rate of 25 mL/min and a heating element temperature of 325 K, 346 K, and 364 K. These trials correspond to the non-perfused trial shown in Fig. 4, allowing direct comparison of perfusion effects.

Comparison of the non-perfused and perfused phantom responses across the 324–364 K temperature range reveals increasingly pronounced perfusion cooling effects at higher probe temperatures. At the lowest probe temperature (324 K, non-perfused compared to 325 K perfused), perfusion produced modest cooling, with the TM1 temperature rise decreasing from 2.3 K to 2.1 K (8.7% reduction) and the TM2 temperature rise from 0.9 K to 0.8 K (11.1% reduction), maintaining a relatively stable thermal gradient of around 1.3–1.4 K. At intermediate temperatures (344 K non-perfused vs 346 K perfused), despite the 2 K higher probe temperature in the perfused trial, TM1 showed only a marginal increase from 4.0 K to 4.2 K. In comparison, TM2 increased from 1.9 K to 2.1 K, suggesting perfusion effectively counteracted the additional heating, with the thermal gradient remaining at approximately 2.1 K in both cases.

The most dramatic perfusion effects emerged at the highest temperatures tested (362 K non-perfused v 364 K perfused), where despite the 2 K higher probe temperature, perfusion reduced TM1 temperature rise from 6.0 K to 4.8 K (20% reduction) and dramatically suppressed TM2 from 3.0 K to 1.5 K (50% reduction), actually increasing the thermal gradient from 3.0 K to 3.3 K. This temperature-dependent enhancement of perfusion cooling, particularly the preferential cooling at the downstream TM2 location, which consistently showed approximately twice the percentage reduction compared to TM1, demonstrates that perfusion channels act as increasingly effective thermal sinks at higher temperature differentials, fundamentally altering both the magnitude and spatial distribution of heat within the tissue phantom.

#### 3.2.2 Combined Effects of Heating Element Temperature and Perfusion

**Figure 6:**
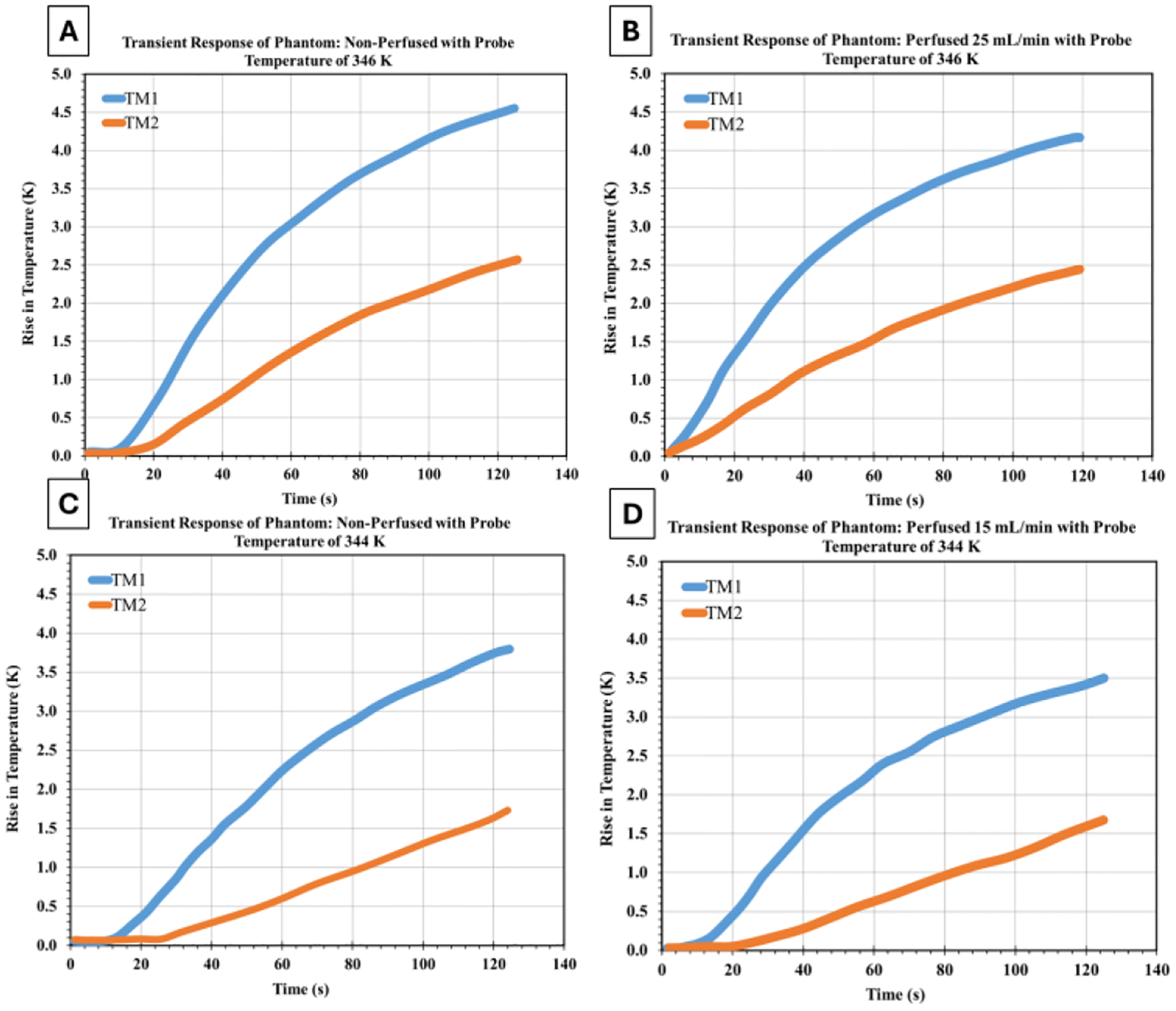
Comparative thermal response of tissue-mimicking phantoms under non-perfused and perfused conditions at probe temperatures of 346 K (A, B) and 344 K (C, D). Left panels (A, C) show non-perfused phantoms exhibiting unimpeded heat accumulation, while right panels demonstrate the cooling effect of perfusion at 25 mL/min and 15 mL/min.

Figure 6 compares phantom responses with and without perfusion at two probe temperatures (346 K and 344 K), which represent typical clinical heating below the tissue coagulation threshold. Without perfusion (Figures A and 6C), the phantoms show standard diffusion-dominated heating. Temperature rises at both measurement points (TM1 and TM2) follow exponential patterns during heating. The temperature gradient between these points reflects pure conductive heat transfer through the polyacrylamide matrix. Perfusion changes these thermal dynamics significantly. At 346 K with 25 mL/min flow (Figure 6B), the perfusion channels actively remove heat, reducing temperature rises at both points.

The cooling effect is spatially dependent—the downstream location (TM2) shows about twice the temperature reduction compared to the upstream location (TM1). This asymmetry confirms directional convective heat removal. At 344 K under lower perfusion conditions characterized by a 15 mL/min flow (Figure 6D), cooling remains effective, especially downstream. This indicates that even modest perfusion rates can significantly impact thermal distribution when channels are well-positioned relative to heat propagation. This finding is clinically relevant for understanding thermal protection in compromised myocardium with reduced coronary flow during ablation. The perfusion effect scales with the temperature gradient between the heated tissue and the perfusate, following standard principles of convective heat transfer. Higher temperature differences produce stronger cooling. This suggests that clinical ablation protocols at different power levels will experience varying degrees of perfusion-mediated cooling, requiring adjusted energy delivery strategies based on local blood flow.

## 4 DISCUSSION

### 4.1 Interpretation of Experimental Results

The experimental investigation of heat transfer in tissue-mimicking phantoms has yielded several significant findings regarding the effects of perfusion on temperature distribution during simulated RF ablation.

#### 4.1.1 Spatial Variation in Perfusion Cooling Effects

A key observation from this study is the spatial variation in cooling effects relative to the perfusion channels. The reduction in temperature rise due to perfusion was consistently greater at TM2 (below the perfusion channels) compared to TM1 (above the channels). On average, perfusion reduced the temperature rise by 9.95% at TM1 and 21.78% at TM2.

The observed asymmetric cooling profile stems from directional heat transfer dynamics within the phantom architecture. Perfusion channels function as thermal interceptors, capturing energy along the path of surface-to-depth heat flux. Convective heat removal through these channels consequently attenuates temperature increases in downstream regions relative to the heat source. This finding has important implications for cardiac RF ablation, where the orientation of blood vessels relative to the ablation electrode can significantly influence lesion geometry. Regions of myocardium downstream of blood vessels (regarding heat flow) may experience greater cooling effects, potentially resulting in incomplete lesion formation [9].

#### 4.1.2 Flow Rate Dependence

The experimental results demonstrated a clear relationship between perfusion flow rate and cooling effect, with higher flow rates producing greater temperature reductions. However, this relationship was not linear, with diminishing returns observed at higher flow rates.

This non-linear behaviour can be explained by the convective heat transfer coefficient, which increases with flow rate. For the flow conditions in this study, with 1.0 mm diameter perfusion channels and flow rates of 15–25 mL/min, the Reynolds numbers range from approximately 320 to 530, indicating laminar flow conditions (Re < 2000). In the laminar flow regime with developing thermal conditions, the convective heat transfer coefficient follows the relationship[35]:

$$h\propto Re^{0.33}\propto v^{0.33},$$

where h is the convective heat transfer coefficient, Re is the Reynolds number, and v is the flow velocity. This correlation, derived from the Sieder-Tate equation for laminar flow in tubes with developing thermal profiles, provides a more accurate representation than the turbulent flow correlation (Re^0.8^) for the specific flow conditions in our perfusion channels [35].

As the flow rate increases, the convective heat transfer coefficient also increases, but at a decreasing rate. Additionally, as more heat is removed from the phantom, the temperature difference between the phantom and the perfusion fluid decreases, further reducing the driving force for heat transfer.

These experimental observations correlate with established clinical experiences during cardiac RF ablation procedures. In clinical settings, heightened regional blood flow is known to diminish lesion formation, often necessitating adjusted energy delivery protocols—either through increased power output or extended application duration—to achieve successful therapeutic endpoints [20].

#### 4.1.3 Interaction with Heating Intensity

The experimental results revealed that the cooling effect of perfusion becomes more pronounced at higher heating intensities. This can be attributed to the temperature-dependent nature of convective heat transfer, where the rate of heat transfer is proportional to the temperature difference between the solid and fluid:

$$q=hA\left(Ts-Tf\right),$$

where q is the heat transfer rate, h is the convective heat transfer coefficient, A is the surface area, T_s_ is the solid temperature, and T_f_ is the fluid temperature.

As the heating element temperature increases, the temperature gradient within the phantom also increases, leading to higher phantom temperatures near the perfusion channels. This larger temperature difference between the phantom and perfusion fluid results in more efficient heat transfer and a more pronounced cooling effect.

This finding has implications for high-power, short-duration RF ablation protocols, which have gained popularity in clinical practice [24]. These protocols may be particularly susceptible to perfusion cooling effects, potentially requiring adjustments in energy delivery parameters to achieve consistent lesion formation.

### 4.2 Comparison with Theoretical Models and Previous Studies

The experimental results from this study provide valuable data for validating theoretical models of bioheat transfer in perfused tissues. The observed reduction in temperature rise due to perfusion is consistent with predictions from the Pennes bioheat equation, which models perfusion as a heat sink term proportional to the temperature difference between tissue and blood [10].

The estimated increase in effective thermal conductivity due to perfusion (11–18% for flow rates of 15–25 mL/min) is comparable to values reported in previous studies, such as those by Valvano et al. [34] reported 10–30% increases in effective thermal conductivity of perfused liver tissue, depending on perfusion rate.

The spatial variation in cooling effects observed in this study aligns with more sophisticated vascular models, such as the Weinbaum-Jiji equation [16], which accounts for the directional effects of blood vessels on heat transfer. These models predict asymmetric temperature distributions around blood vessels, with greater cooling effects in regions downstream of the heat flow path.

Previous experimental studies using perfused tissue phantoms have reported similar findings. Mikhail *et al*. [30] observed reductions of 15–25% in lesion volume during RF ablation of perfused cardiac phantoms compared to non-perfused controls. Similarly, González-Suárez *et al*. [39] reported that perfusion could reduce maximum tissue temperature by up to 10°C during RF ablation at 30W, consistent with the temperature reductions observed in our study when scaled for power differences.

The focus of this specific component of our work was to better understand the effect of perfusion on tissue temperature distribution and specifically quantify tissue temperature in response to the presence and various extents of perfusion. As such, the current study focused on experimental characterization of perfusion effects by conducting transient tissue temperature measurements at specific locations in tissue-emulating phantoms featuring different perfusion scenarios. Complementary numerical work using computational fluid dynamics (CFD) has been conducted in an earlier preliminary study [36] aimed at serving as a first-order attempt at numerically replicating some of these experimental findings. This work employed ANSYS Fluent, incorporating the Pennes bioheat equation, to model heat transfer in perfused tissue phantoms and provided a first-order comparison of the model-predicted temperature profiles and the experimental temperature measurements. The end-ablation temperature model predictions showed agreement with the experimental temperature measurements, particularly in capturing perfusion-induced cooling at different depths within the phantom. Further computational studies are needed as part of our future work to extend these models to incorporate experimental data, thereby improving the accuracy and realism of lesion characterization and prediction algorithms.

### 4.3 Implications for Cardiac RF Ablation

The findings of this study have important implications for cardiac RF ablation procedures. First, the pronounced cooling effect of perfusion, especially at higher heating intensities, underscores the need to include perfusion in lesion prediction models; neglecting it may lead to overestimating lesion size and incomplete ablation. Second, the observed spatial variation in cooling suggests that the orientation of the electrode relative to coronary vessels can affect lesion geometry, providing valuable guidance for optimizing electrode placement and energy delivery during procedures. Third, the non-linear relationship between heating intensity and perfusion cooling indicates that adjusting power delivery in high-perfusion regions could help achieve more consistent lesion formation. Finally, since clinical RF ablation often uses electrode irrigation, understanding the interplay between tissue perfusion and external saline flow can inform the development of improved irrigation strategies that better account for the complex thermal environment during ablation.

### 4.4 Limitations and Future Directions

While this study offers valuable insights into how perfusion affects heat transfer in tissue-mimicking phantoms, several limitations should be noted that constrain the direct translation of our findings to clinical scenarios. The phantom’s parallel perfusion channels (1.0 mm diameter, 5 mm spacing) represent a simplified version of the coronary vasculature. In contrast, absolute myocardial perfusion involves a much more complex and variable vessel network ranging from 3–4 mm epicardial vessels to 100 μm capillaries. Additionally, the experiments employed constant flow rates (15–25 mL/min), whereas actual coronary blood flow is pulsatile and varies three to five times with the cardiac cycle. The phantom material, though chosen to match the thermal properties of cardiac tissue, does not capture the anisotropic and heterogeneous nature of real myocardium. The use of a heated element, rather than actual RF energy, means the study focuses solely on thermal effects and does not account for the electromagnetic aspects of clinical ablation. Finally, the temperature range of these experiments was lower than that typically reached during clinical RF ablation. These simplifications imply that our emulated cooling behavior represents idealized conditions, whereas actual clinical cooling may vary substantially due to local vascular architecture and flow dynamics.

Lastly, the uncertainty related to thermocouple placement, which was mentioned earlier in this study, might become more relevant when comparing experimental measurements to model predictions. To allow for accurate comparison, it is important to ensure that the tissue locations sampled by the model match the locations of the thermocouples during the experiments. This limitation underscores the need for improved techniques to track or confirm thermocouple placement, and, therefore, future studies, which will also incorporate numerical model prediction and lesion characterization, will include multiple thermocouples deployed at specific positions verified using image-guided placement, to ensure sufficiently accurate thermocouple deployment and minimize the effect of thermocouple positioning uncertainty.

Future research could address these limitations by developing phantoms with more realistic, heterogeneous structures, integrating thermochromic materials to visualize temperature and lesion formation, and using actual RF energy sources. Studies could also explore the effects of pulsatile flow and varying perfusion rates and use the experimental data to validate and refine computational models for improved lesion prediction.

## 5 CONCLUSION

This study has provided a comprehensive experimental investigation of perfusion effects on heat transfer in tissue-mimicking phantoms for cardiac RF ablation. Building upon a robust and statistically validated methodology, the results demonstrate that simulated blood perfusion significantly reduces temperature rise, with a markedly greater effect observed at measurement points distal to the perfusion channels (average 21.78% reduction) compared to proximal points (average 9.95% reduction). This spatial variation in the heat sink effect was consistently observed across multiple flow rates and heating intensities.

The cooling effect of perfusion increased with flow rate in a non-linear manner, exhibiting diminishing returns at higher flows due to a reduced temperature gradient driving force. Furthermore, a critical finding was the non-linear interaction between heating intensity and perfusion cooling, where the effect became substantially more pronounced at higher temperatures. This was quantified by an increase in the phantom’s effective thermal conductivity of 11–18% for flow rates of 15–25 mL/min.

While this controlled model, utilizing a simplified parallel channel geometry, provides fundamental insights, it also highlights the complexity associated with realistic, *in vivo* conditions. The findings have essential implications for clinical RF ablation, underscoring that the orientation of ablation electrodes relative to coronary vessels can critically influence lesion geometry and procedural success. This work successfully bridges a gap between theoretical bioheat models and experimental validation, offering a valuable dataset for refining computational predictions. The experimental platform and methodology established here lay a rigorous groundwork for future research into the intricate relationships between RF energy delivery, tissue characteristics, and blood perfusion. Immediate next steps may include integrating real RF energy sources and more anatomically realistic phantom designs to enhance the clinical translatability of the results.
